# Healthy Behavior and Environmental Behavior Correlate with Bicycle Commuting

**DOI:** 10.3390/ijerph19063318

**Published:** 2022-03-11

**Authors:** Qiang Liu, Toshiaki Yamada, Hang Liu, Li Lin, Qiaoling Fang

**Affiliations:** 1 Institute of Policy Research, Kumamoto 860-0806, Japan; 2 Faculty of Advanced Science and Technology, Kumamoto University, Kumamoto 860-8555, Japan; 3 Department of Contemporary Sociology, Shiseikan University, Hagi 758-8585, Japan; sundaysohlyou@gmail.com; 4 School of Tourism and Urban-Rural Planning, Zhejiang Gongshang University, Hangzhou 310018, China; liuhang0611@gmail.com; 5 Zhejiang Development & Planning Institute, Hangzhou 310030, China; linpopo4768@163.com; 6 Graduate School of Design, Kyushu University, Fukuoka 815-8540, Japan; fangql0101@outlook.com

**Keywords:** bicycle commuting, healthy behavior, environmental behavior, factor analysis, binary logistic regression

## Abstract

Previous studies have identified that environmental awareness correlates with the choice of bicycle travel. However, few studies have considered the relationships with different types of healthy behaviors and environmental behaviors. This study examined the relationships between several healthy and environmental behaviors and the choice of bicycle commute using survey data. A total of 803 residents participated in this questionnaire survey. Using factor analysis, we constructed latent factors of healthy behaviors and environmental behaviors. Using a binary logistic regression model, we examined the relationship between latent factors and cycling usage, controlling for demographic characteristics. Factor analysis revealed three latent factors of healthy behaviors: “healthy diet”, “avoiding tobacco or overdrinking”, and “physical activity”. The latent factors of environmental behaviors were as follows: “household behavior” and “purchasing behavior”. The results showed that “avoiding tobacco or overdrinking”, “physical activity” and “purchasing behavior” correlated positively with bicycle commuting. Differences were also observed in relation to demographic characteristics.

## 1. Introduction

Our reliance on cars has aggravated traffic congestion, traffic related crash, and environmental loads in cities [1]. To ease traffic and environmental problems, recent research has increasingly emphasized the importance of cycling [2,3]. Although cycling has risks (e.g., injury and environmental pollutant exposure) for cyclists, it has been found that the benefits surpass these risks [4,5]. Cycling is usually an efficient mode of transportation that could mitigate traffic congestion by reducing car traffic [6]. Cycling is also regarded as a mode of sustainable transportation that could provide benefits at individual and social levels. At the individual level, there are significant health benefits for cycling, including weight control and decreased cardiovascular disease [7]. Palencia et al. [8] also have proved that commuting by bicycle could reduce the risk of stress. At the social level, environmental benefits for cycling have been demonstrated, primarily because it can reduce the emission of toxic gas and harmful particles [9]. Various research has been conducted to detect how to increase cycling commute [10,11,12].

Generally, cycling usage may be correlated with factors such as sociodemographic characteristics, policy support, cycling infrastructure, and awareness [13]. The main scope of our research is the latter with an emphasis on health behavior and environmental behavior. As cycling usage with different motivations is characterized by different patterns of action, we focus our study on commuting. In this paper, we tried to identify the relationship between healthy and environmental behavior and bicycle commute.

Previous studies indicated that sociodemographic characteristics correlate with cycling usage. Table 1 summarizes some studies. Porter et al. [11] performed a cross-sectional study using U.S. adults as a sample and found that males are more likely to use bicycles than females. However, Garrard et al. [14] reported that females are more likely to use bicycles in regions with a low share of bicycle trips. Hunter et al. [15] reported that nationality has a significant relationship with bicycle commute in Barcelona. Dill et al. [16] performed a survey in Portland and found age to be negatively correlated with cycling. Miller and Handy [17] indicated that age had no significant relationship with bicycle commuting in Davis. Heinen et al. [18] reported that bicycle commute is strongly linked to personal and household characteristics based on various studies.

Several studies have demonstrated that policy interventions relative to cycling also have an important influence on cycling usage [19,20,21,22]. Pérez et al. [23] conducted a pre-post evaluation study and reported that policies promoting cycling increased the number of cycling usage from 2009 to 2013 in Barcelona. Braun et al. [24] used data from a travel survey in Barcelona and indicated that policies that combined public transport and cycling could be useful strategies for increasing bicycle commutes in short-term periods. Caulfield [25] examined if policy interventions, such as bicycle-purchasing assistance schemes, could have a positive impact on bicycle commuting in Dublin. They found that the cycling rates of commuters increased after these policies. However, policy interventions alone cannot ensure long-term improvements in cycling usage [3].

Some previous studies have indicated that improving existing cycling infrastructure, such as bike lanes and separated bike paths, could effectively increase bicycle commute [26,27]. Kelarestaghi et al. [28] conducted a survey on college campuses and found that the quality cycling infrastructure could promote cycling usage. In addition, the advancement of cycling infrastructure has a positive impact on the perceptions of safety and comfort [29,30]. Greater bikeway densities and street network connectivity induce greater cycling usage as a mode of transportation [31,32]. However, due to space restrictions, the roads of many cities in Japan are narrow, which is quite difficult to improve cycling infrastructure [33].

On the other hand, recent studies have shown that attitudes or perceptions have been known as significant variables relating to the intention to bicycle commuting [34,35]. In addition, health awareness and environmental awareness also have a statistically significant relationship with bicycle commute [36,37,38,39]. Börjesson and Eliasson [6] used two established Multi-Attribute Decision-Making approaches to study the motivators associated with cycling usage. They indicated that health awareness is perceived as an important determinant of cycling usage; however, it does not explain the meaning or standard to define health awareness. Gatersleben and Appleton [40] conducted a study amongst university staff and students and found that personal attention to health is highly related to cycling for commuting purposes. Heinen et al. [18] reported that commuters who are more concerned about their health would cycle more. However, these previous studies have only focused on health awareness towards physical exercise or fitness.

Environmental awareness in most of the previous studies also remains incomprehensive. Kumagai and Managi [41] tested the relationship between environmental behaviors that can be taken at home and travel mode choice. They indicated that environmental activities such as recycling activities positively correlated with bicycle commute in Beijing. Majumdar et al. [38] indicated that environmental awareness of air pollution is perceived to be important for bicycle trips in two typical Indian cities. Lind et al. [42] conducted a questionnaire survey among six cities in Norway and applied structural equation modeling to explain travel mode change. The results suggested that individuals who have a high awareness of the responsibility for environmental problems would be more likely to use bicycles. Li et al. [37] used K-means to cluster bicycle commuting into six segments. The commuter segments with high environmental awareness toward air quality also had a high willingness to use bicycles. However, most of these previous studies have only focused on one kind of environmental awareness such as carbon emission or energy use.

**Table 1:** Summary of factors in previous studies.

Factors	First Author	Year	Country	City	Data Collection
Gender	Porter [11]	2018	United States	-	National survey
	Garrard [14]	2008	Australia	Melbourne	Field survey
Age	Dill [16]	2007	United States	Portland	Preference survey
	Miller [17]	2008	United States	Davis	Preference survey
Household type	Ryley [43]	2006	Scottish	Edinburgh	National survey
	Liu [44,45]	2017	China	Tianjin	Preference survey
Employment status	Fu [13]	2017	United States	Salt Lake	Preference survey
Home district	Damant-Sirois [45,46]	2015	Canada	Montreal	Preference survey
Environmental factor	Kumagai [41]	2019	Japan, China, Singapore	Tokyo, Beijing, Signapore	Preference survey
	Li [37]	2013	China	Nsnjinh	Preference survey
Healthy factor	Gatersleben [40]	2007	United Kingdom	Guildford	Preference survey
	Heinen [18]	2010	Netherlands	Delft	Literature review

The above findings could strongly support our research. We also noticed that they mainly focused on one kind of health awareness or environmental awareness. While a limited number of studies focused on both health and environmental factors [39,44,46], they are concerned with the effect of the sum of health, environmental, and other awareness. Furthermore, little research has analyzed different types of health and environmental awareness, which indicates that they may not be well explored. Hence, to better understand the effect of health and environmental factors on bicycle commute, we propose a model combining health behavior and environmental behavior. We further refined each behavior to different types and find that environmental behavior following a desire to save money has no significant relationship with bicycle commuting.

The purpose of this research is to identify the relationships between different types of healthy and environmental behavior and the choice of cycling usage, controlling for demographic characteristics. Since commuting accounts for a large proportion of daily travel demand, this paper focuses on bicycle commute (work/study) trips. We also clearly defined the health behavior and environmental behavior and further refined the factors from different types (Section 2.1). In this paper, data from a survey of adults were mainly used to (1) construct the latent behavioral factors of healthy behaviors and environmental behaviors and (2) identify the relationships between latent behavioral factors and bicycle commuting. We conducted factor analysis to construct the latent behavioral factors of healthy behaviors and environmental behaviors. A binary logistic regression model was utilized to explain the relationship between behavioral variables and choice of cycling usage, controlling for demographic characteristics.

## 2. Materials and Methods

### 2.1. Theoretical Framework

**Figure 1:**
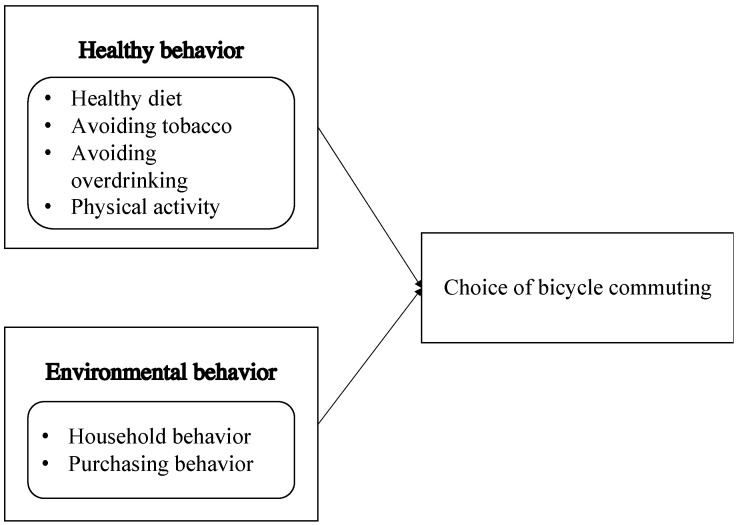
The research framework.

We presume that healthy behaviors and environmental behaviors may be related to the choice of bicycle commuting. The modeling framework of this research is presented in Figure 1.

Heathy behavior refers to ‘actions and habits that relate to health maintenance, to health restoration and to health improvement’ [47]. Widely studied health behaviors may be classified as healthy diet, avoiding tobacco, avoiding overdrinking, and physical activity [48]. Therefore, we refine healthy behaviors into these four aspects: (a) healthy diet, (b) avoiding tobacco, (c) avoiding overdrinking, and (d) physical activity:

- Healthy diet refers to balanced diet, breakfast, and sufficient vegetable intake.
- Avoiding tobacco refers to not smoking.
- Avoiding overdrinking refers to not excessive alcohol use (binge drinking and heavy drinking).
- Physical activity refers to bodily movement by practicing sport.

The second part is related to environmental behavior that refers to environmentally friendly behavior. Environmental behavior could be more explicitly defined as pro-environmental behaviors that aim to minimize any adverse effects on the availability of materials or energy from the natural environment [49]. Environmental behavior may be classified into private sphere and public sphere [50]. This paper mainly focuses on individual behaviors that have direct environmental consequences, such as the purchase, utilization, and disposal of household and personal products. It may be subdivided into household behavior and purchasing behavior [50]. Therefore, we refine the individual’s environmental behaviors into two aspects: (a) household behavior and (b) purchasing behavior:

- Household behavior refers to water use, energy use, and household waste disposal;
- Purchasing behavior refers to the purchase of personal and household products that are environmentally friendly in their use and production processes.

### 2.2. Data Collection

This paper used sample data from a citizen survey that was conducted on the citizens’ quality of life in Kumamoto, Japan. Kumamoto is the third largest city on Kyushu Island with a population of 740,000. The survey was conducted by Kumamoto City Hall with questionnaires. The questions in the questionnaire were designed to reflect the quality of life. To develop the survey questionnaire, several similar surveys conducted by other cities were referenced. The questionnaire was assessed by some researchers from local universities. Before questionnaire distribution, a pre-test was conducted to modify the confusing questions from the feedback of the respondent. The final questionnaire included 5 components: a survey of mobility (e.g., mode choice and safety), a survey of living environment (e.g., noise and cleanliness), a survey of public service (e.g., infrastructure and public services), a survey of social welfare (e.g., medical care and educations), and demographic characteristics (e.g., age and gender).

Respondents were randomly selected from the Basic Resident Registration System in Kumamoto city, with eligible individuals aged ≥20 years. The sample distribution was balanced by the proportion of population in 5 districts (center district, east district, west district, south district, and north district). Finally, a total of 5000 respondents were selected and approached by mail. The questionnaires were distributed on 25 November by mail. They were asked to send the questionnaires back before 13 December by using the return envelope enclosed and 1780 were recovered as of 20 December.

The data in this research were obtained from the mobility component. Respondents were asked to answer commuting mode choice and non-commuting mode choice. The respondents who did not answer the mode choice of commuting or were aged >65 years old (retired) were deleted, because this research focuses on commuting trips. According to whether they commute by bicycle, respondents were classified into two categories of travel mode: (1) bicycle and (2) non-bicycle. Note that responses with missing answers were also deleted, and a final sample included 803 questionnaires.

Details of the questionnaire used in this study involved four parts: (1) commuting modes: bicycle and non-bicycle; (2) demographic characteristics of the respondent: gender, age, household type, employment status, home ownership, and home district; (3) healthy behaviors: questions on healthy diet, avoiding tobacco, and avoiding overdrinking and physical activity; and (4) environmental behaviors: questions on household behavior and purchasing behavior. Table 2 shows the demographic characteristics of respondents. In the survey, there were seven questions on healthy behaviors and environmental behaviors each (Table 3). Respondents were asked to choose the fourteen behaviors they have, where “1” means “yes” and “0” means “no”.

### 2.3. Statistical Analysis

We used factor analysis to construct the latent behavioral factors of healthy behaviors and environmental behaviors. Then, a binary logistic regression model was used to explain the relationship between behavioral variables and bicycle commuting, controlling for demographic characteristics. The overview of factor analysis and the binary logistic regression model are briefly introduced in this section.

#### 2.3.1. Factor Analysis

The behavior responses were analyzed using factor analysis. Factor analysis is a statistical technique used to group variables together in terms of correlation among observed variables [52]. The purpose of factor analysis is variable reduction and maintenance of the explanatory power of the original variables. In this study, exploratory factor analysis was conducted to drop survey responses and confirm the latent structures underlying behavior responses.

A total of 14 survey responses in Table 3 were tested by factor analysis. The Kaiser–Meyer–Olkin (KMO) and Bartlett’s test of sphericity were used to confirm that the sample is suitable. For the rotation method, we used varimax rotation to allow factors to correlate [53]. The authors selected factors with eigenvalues greater than 1 to ensure they could contribute to the explanation of variances in the variables. The factor score was also used for subsequent analysis with the regression method.

#### 2.3.2. Binary Logistic Regression

In this study, since the choice of bicycle commuting is a binary variable, we used a binary logistic regression model to explore the relationships between dependent variable and independent variables. The suggested factors by exploratory factor analysis are explanatory variables, such as factors reflecting healthy behavior and environmental behavior. Additionally, demographic characteristics, such as gender, age, household type, employment status, home ownership, and home district, are regarded as covariates. The variables that have no statistically significant relationship with cycling usage were removed. The binary logistic regression was performed by IBM SPSS version 25.

## 3. Results

### 3.1. Factor Analysis

The results of exploratory factor analysis are shown in Table 4. The variables with low communality (factor score < 0.45) are not reported. KMO is 0.727 and the Bartlett’s test of sphericity is significant, which suggests that our sample is sufficient for the analysis. Finally, a total of 14 variables were identified to form five factors (Table 4).

The healthy behavioral variables were refined into three factors: (1) “healthy diet (HD)”, related to Q1–Q3; (2) “avoiding tobacco or overdrinking (ATO)”, related to Q4–Q5; and (3) “physical activity (PA)”, related to Q6–Q7. The environmental behavioral variables were refined into two factors: (1) “household behavior (HB)”, related to Q8–Q11; and (2) “purchasing behavior (PB)”, related to Q12–Q14.

### 3.2. Binary Logistic Regression

The results of binary logistic regression are shown in Table 5. The dependent variable was “the choice of bicycle commuting” (1 if bicycle and 0 if non-bicycle). The factors that reflect healthy behaviors are healthy diet, avoiding tobacco or overdrinking, and physical activity. The factors that reflect environmental behaviors are household behavior and purchasing behavior. The bottom rows of the table show that Cox-Snell R^2^ is 0.126 and Nagelkerke R^2^ is 0.182. The Hosmer–Lemeshow test is not significant. ATO, PA, and PB all have a significant relationship with cycling usage and an odds ratio above 1, indicating that a unit change on these factors could increase cycling usage. The demographic characteristics (gender, employment status, and district) are also significant for cycling usage. The other variables with no significant relationship are not reported in the table.

## 4. Discussions

The variables that affected bicycle commuting included avoiding tobacco or overdrinking, physical activity, purchasing behavior, gender, employment status, and home district. Regarding demographic characteristics, the results indicate that males are significantly more likely than females to use bicycles (OR = 1.403). This finding is consistent with most former research studies such as Sallis et al. [12] and Porter et al. [11], perhaps because females are more risk averse than males [54]. However, it is in contrast to the findings of Garrard et al. [14], who indicated that females contribute more to the use of bicycles in regions with a low share of bicycle trips. We also found that age had no significant relationship with cycling usage which is consistent with Miller and Handy [17] who conducted a study in a bicycle-friendly community. This finding is in contrast with Dill et al. [13] who conducted a survey in Portland and found age to be negatively correlated with cycling. Employment status had a significant relationship with cycling usage, which might be because employment status indirectly impacts travel mode through income [55]. The respondents’ home district is significantly related to cycling usage; the people living in the Center district (OR = 4.795) and East district (OR = 4.366) were especially likely to use bicycles. This might be because the Center district and East district are urban areas with quality cycling infrastructure and greater street network connectivity than the other districts [56]. Moreover, household type has no significant relationship with bicycle commuting in this study, which is in contrast with the results of Kumagai and Managi [41] who conducted a survey in Beijing and Singapore.

The healthy behavioral variables were refined into three factors: healthy diet (HD), avoiding tobacco or overdrinking (ATO), and physical activity (PA). ATO and PA are variables that significantly relate to bicycle commuting, while HD was not. ATO (β = 0.169) is positively correlated with bicycle commuting, which indicates that adults who are concerned about being healthy are more likely to commute by bicycle. PA (β = 0.442) also has a positive correlation, which indicates that adults who are concerned about physical fitness are more likely to commute by bicycle, which is consistent with Sisson and Tudor-Locke [57]. Since cycling is a form of transportation that requires physical activity, it is readily comprehensible that people who practice sports would be more likely to commute by bicycle. However, concerns about personal diet do not correlate with bicycle commute. This might be because, depending on the type of diet, the focus is regular and habitual food intake. In contrast, ATO and PA are spontaneous actions for health, making them more proactive.

The environmental behavior variables were refined into two factors: household behavior (HB) and purchasing behavior (PB). HB is not significantly related to bicycle commuting. This might be because the main purpose of household behaviors is usually to reduce household expenditure. Stern [50] also put forward a similar point of view that some environmental behaviors may follow from a desire to save money. These results are in contrast with Kumagai and Managi [41], who indicated that environmental behaviors taken at home have a significant relationship with bicycle commuting in Beijing. However, PB (β = 0.22) is positively correlated with bicycle commuting. Unlike household behaviors, purchasing behaviors may cost more because ecofriendly products are more expensive at the moment of payment [58]. Purchasing behavior may mainly follow from environmental intent while household behavior may mainly follow from nonenvironmental concerns. In this study, purchasing behaviors may be a better indicator of adults that are concerned about the environment than household behaviors.

Previous studies mainly suggested that environmental awareness has a positive relationship with cycling usage [12,36]. However, our findings emphasized that different types of healthy behaviors and environmental behaviors have different relationships with bicycle commute. Media campaigns may be necessary to enhance ATO, PA, and PB behaviors of the general public. Mozaffarian et al. [59] have proved that media strategies such as television, radio, newspaper, billboard, or transit ads could improve behavior. Overall, these findings could provide insight for policy makers and planners: media focused on physical activity, avoiding tobacco or overdrinking, and environmentally friendly purchasing behaviors may be effective in encouraging an increase in the corresponding behavior of using bicycles.

## 5. Conclusions

In this study, we conducted a study on the relationships between healthy and environmental behavior and bicycle commuting in Kumamoto City. This study explored healthy behaviors and environmental behaviors using factor analysis and then analyzed these factors, along with demographic characteristics, for relationships with bicycle commuting using binary logistic regression models.

The results suggested that demographic characteristics of individuals are significantly related to bicycle commuting. In this paper, we also analyzed the relationship between several behaviors and bicycle commuting. The results indicated that “avoiding tobacco or overdrinking”, “physical activity”, and “purchasing behavior” are positively correlated with using bicycles. It should be noted that this may also be vice versa, for active commuting by bicycle is can also impact health [60].

As suggested by previous research, environmental awareness and health awareness, only toward one type, have significant relationships with cycling usage [6,11,12,36,41]. The main contributions of our research are as follows: (1) Our research underlined the importance of focusing on different types of environmental behaviors and healthy behaviors that have been clearly defined; (2) our research also demonstrated that not all the types of environmental behaviors or healthy behaviors can be considered correlated with bicycle commuting, which has been ignored by most previous research; and (3) the findings of this study could help policy makers and planners develop more focused policies. We suggest offering appropriate bicycle policies that target users who engage in “avoiding tobacco or overdrinking”, “physical activity”, or “purchasing behavior” among commuters. The results in this paper could also provide a reference for researchers to explore the correlation between different types of healthy and environmental behaviors and bicycle commute.

## Figures and Tables

**Table 2:** Demographic characteristics of respondents.

Variable	Attribute	Total (*n* = 803)	
		Frequency	Proportion
Gender	Female	448	55.8%
	Male	355	44.2%
Age	20–29	78	9.7%
	30–39	138	17.2%
	40–49	198	24.7%
	50–59	163	20.3%
	≥60	226	28.1%
Household type	Single	110	13.7%
	Couple no children	219	27.3%
	Couple with children	387	48.2%
	All other families	87	10.8%
Employment status	Employed	507	63.1%
	Student ^a^	26	3.2%
	Unemployed ^b^	204	25.4%
	Other	66	8.2%
Home ownership	Rents	303	37.3%
	Owns	475	59.2%
	Other	25	3.1%
Home district	Center district	204	25.4%
	East district	217	27%
	West district	95	11.8%
	South district	129	16.1%
	North district	158	19.7%
Bicycle commuting	Bicycle	224	27.9%
	Non-bicycle	579	72.1%

^a^ University student. ^b^ Part-time workers.

**Table 3:** Frequency and proportion of the healthy and environmental behavior.

Questions	Content	Frequency (Proportion)
Healthy behavior		
Q1	I have a balanced “Japanese diet” centered on rice ^a^ (yes = 1)	279 (34.7%)
Q2	I eat breakfast everyday (yes = 1)	529 (73.7%)
Q3	I eat vegetables more than twice a day (yes = 1)	374 (41.7%)
Q4	I don’t over drink (yes = 1)	392 (48.8%)
Q5	I don’t smoke (yes = 1)	548 (68%)
Q6	I practice sports for more than 30 min every time (yes = 1)	294 (36.3%)
Q7	I practice sports more than three times a week (yes = 1)	214 (26.6%)
Environmental behavior		
Q8	I don’t leave the lights/TV on (yes = 1)	619 (77.1%)
Q9	I turn the faucet on then off frequently ^b^ (yes = 1)	564 (70.2%)
Q10	I set the air conditioner on the ideal temperature to avoid waste (yes = 1)	588 (73.2%)
Q11	I always sort out my garbage (yes = 1)	550 (68.5%)
Q12	I don’t use plastic shopping bags (yes = 1)	464 (57.8%)
Q13	I usually purchase recycled products (yes = 1)	109 (13.6%)
Q14	I usually choose eco-friendly products when shopping (yes = 1)	173 (21.5%)

^a^ Koga et al. [51] indicated that Japanese diet centered on rice may improve mental health. ^b^ To avoid wasting water.

**Table 4:** The results of exploratory factor analysis.

Questions	Factors				
	Healthy Diet	Avoiding Tobacco or Overdrinking	Physical Activity	Household Behavior	Purchasing Behavior
Q1	0.664				
Q2	0.687				
Q3	0.633				
Q4		0.802			
Q5		0.782			
Q6			0.91		
Q7			0.917		
Q8				0.8	
Q9				0.757	
Q10				0.706	
Q11				0.504	
Q12					0.669
Q13					0.51
Q14					0.749

**Table 5:** Binary logistic regression results.

Variables	Cycling Usage			
	Coeff	*p*-Value	OR ^a^	95% CI ^a^
Constant	−0.969 *	0.05	0.379	
Healthy diet	−0.062	0.466	0.94	0.795–1.111
Avoiding tobacco or overdrinking	0.169 *	0.058	1.184	0.994–1.41
Physical activity	0.442 ***	0.000	1.555	1.318–1.863
Household behavior	0.081	0.340	1.085	0.918–1.282
Purchasing behavior	0.22 ***	0.009	1.246	1.056–1.47
Gender				
Male	0.338 *	0.06	1.403	0.986–1.995
Female	0		1	
Employment status				
Employed	−1.4 ***	0.002	0.247	0.102–0.594
Student	−1.163 **	0.027	0.312	0.112–0.875
Unemployed	−1.226 ***	0.009	0.293	0.117–0.733
Other	0		1	
Home district				
Center district	1.568 ***	0.000	4.795	2.675–8.597
East district	1.474 ***	0.000	4.366	2.442–7.806
West district	0.617 *	0.097	1.854	0.894–3.843
South district	0.863 **	0.01	2.371	1.226–4.585
North district	0		1	
Cox-Snell R^2^	0.126			
Nagelkerke R^2^	0.182			
Hosmer-Lemeshow test	3.994			

^a^ ORs and 95% CIs adjusted for all other variables. * Significant at the 0.1 level. ** Significant at the 0.05 level. *** Significant at the 0.01 level.

## Data Availability

Data sharing not applicable.
